# Effectiveness comparisons of traditional Chinese medicine on treating diabetic nephropathy proteinuria: A systematic review and meta-analysis

**DOI:** 10.1097/MD.0000000000017495

**Published:** 2019-10-25

**Authors:** Lin Wang, Ya-Hui Wang, Xian-Hui Zhang, Xue-Lian Yang, Hui-Li Wei, Zhi-Chao An, Bo-Rui Yu, Da-Qing Du, Yan Guo, Hong-Fang Liu

**Affiliations:** Nephrology Department, Dongzhimen Hospital Affiliated to Beijing University of Chinese Medicine, Beijing, China.

**Keywords:** diabetic nephropathy proteinuria, protocol, systematic review, traditional Chinese medicine

## Abstract

**Background:**

Diabetic nephropathy (DN) is one of the microvascular complications of diabetes mellitus. Proteinuria is the most important clinical feature of DN and an independent risk factor for the progression of DN. Therefore, reducing urinary protein is the primary goal of DN treatment. Traditional Chinese medicine (TCM) has long been widely used in the treatment of DN. Therefore, this paper conducted a meta-analysis of the clinical efficacy of TCM in the treatment of DN proteinuria, to comprehensively analyze the role of TCM in the treatment of DN.

**Methods:**

We will search for PubMed, Cochrane Library, AMED, EMbase, WorldSciNet, Nature, Science online and China Journal Full-text Database, China Biomedical Literature CD-ROM Database, and related randomized controlled trials included in the China Resources Database. The time is limited from the construction of the library to September 2019. We will use the criteria provided by Cochrane 5.1.0 for quality assessment and risk assessment of the included studies, and use the Revman 5.3 and Stata13.0 software for meta-analysis of the effectiveness, recurrence rate, and symptom scores of DN proteinuria.

**Trial registration number:**

PROSPERO CRD42019139707.

## 1 Introduction

Diabetic nephropathy (DN) is one of the most common and serious chronic complications of diabetes mellitus (DM),^[1]^ and proteinuria is the most important clinical feature of DN and an independent risk factor for DN progression.^[2]^ The production of microalbuminuria can increase the all-cause mortality of DM patients.^[3]^ At present, the treatment of DN is based on lifestyle changes and eating habits, control of blood sugar, blood pressure, and correction of dyslipidemia.^[4]^ In recent years, although many studies have confirmed that drugs including vitamin D,^[5]^ immunosuppressant tacrolimus^[6,7]^ have a certain effect on reducing DN proteinuria and delaying the progression of the disease, but it is still recommended in clinical practice. Only ACEI/ARB drugs. The application of ACEI/ARB drugs has its limitations. Studies have shown that ACEI/ARB is beneficial for diabetic patients with hypertension with GFR <60 mL/min/1.73 m^2^ and UACR ≥300 mg/g Cr, which can delay their chronic kidneys disease progress. However, for patients with normal blood pressure, ACEI/ARB cannot prevent the occurrence of kidney damage caused by diabetes.

Traditional Chinese medicine (TCM) treatment of DN proteinuria is clinically effective, and a variety of TCM extracts or combination preparations have been confirmed to have a clear renal protective effect.^[8–11]^ The theory of TCM believes that the location of DN is mainly in the kidney, the deficiency of kidney and the stagnation of renal collateral are the key to the pathogenesis of DN.^[12–16]^ Yin deficiency and heat, qi stagnation and blood stasis is its main pathogenic factor. Through the application of TCM in the treatment of DN proteinuria's unique diagnosis and treatment system, clinical efficacy is significant. Modern research has shown that effective active ingredients in TCM can improve the blood supply of peripheral blood vessels and achieve therapeutic purposes.^[17,18]^ Through the action mechanism of multi-faceted and multi-target, TCM regulates the body function as a whole and has unique advantages in the treatment of DN proteinuria. After preliminary search and analysis of database, we found that the frequency of randomized controlled trials (RCTs) of TCM treatment in DN proteinuria has been showing an increasing trend.^[19]^ Previous clinical trials have shown that TCM could ameliorate pain and improve the quality of lives in patients who suffer from DN proteinuria, and these effects are sustained.^[17,20]^ However, due to the limitation of the scale and sample size of the clinical centers, the current level of evidence-based medical evidence is still not sufficient. Therefore, we hope to evaluate the efficacy and safety of TCM in treating DN proteinuria by using meta-analysis, which aim to provide sufficient evidence for its clinical application.

## 2 Methods

This systematic review protocol has been registered on PROSPERO as CRD42019139707. (https://www.crd.york.ac.uk/prospero/display_record.php?ID=CRD42019139707). The protocol follows the Cochrane Handbook for Systematic Reviews of Interventions and the preferred reporting items for systematic reviews and meta-analysis protocol (PRISMA-P) statement guidelines. We will describe the changes in our full review if needed.

### 2.1 Inclusion criteria

#### 2.1.1 Types of studies

This study will include all the RCTs that relate to TCM therapy in treating DN proteinuria. For the included trials, the investigators need to precisely report the stochastic methods, TCM treatment details and parameters, diagnostic criteria and efficacy evaluation they based on. No limitation to whether it is published or not. The experiment is limited to humans. Language is limited to Chinese and English.

#### 2.1.2 Types of participants

Participants who were definitely diagnosed with DN proteinuria would be included. The cases which relate to prostatic hyperplasia, prostate cancer or other prostate-related diseases would be excluded. In addition, there are no limitations in region, citizenship, nationality, and source of cases.

#### 2.1.3 Types of interventions

##### 2.1.3.1 Experimental interventions

The drug composition, the dose-specific Chinese medicine preparation or the combined western medicine are used as experimental interventions. Both prescription and Chinese patent medicines will be included. Other TCM treatments such as intravenous medication, acupuncture, and massage will be limited.

##### 2.1.3.2 Control interventions

As for the control interventions, who accepted simple western medicine can be used as a control intervention or did not get any treatment as a blank control would be adopted. However, once they had accepted the therapy of TCM, the trials will be rejected.

#### 2.1.4 Types of outcome measures

##### 2.1.4.1 Primary outcomes

Primary outcomes included 24-hour urine protein quantitation, urinary albumin excretion rate, fasting blood glucose, glycosylated hemoglobin, and total effective rate.

##### 2.1.4.2 Secondary outcomes

The second outcome measure is based on TCM syndrome evaluation criteria.

(1) Healing: the clinical symptoms and signs of TCM disappear or almost disappear, and the syndrome score is reduced by ≥90%;
(2) Significant effect: the clinical symptoms and signs of TCM are obviously improved, and the syndrome score is reduced by ≥60%;
(3) Effective: Chinese medicine clinical symptoms and signs have improved, syndrome scores decreased by <60%, but ≥30%;
(4) Invalid: the clinical symptoms and signs of TCM were not improved, even worse, and the syndrome score was reduced by <30%.

Integral variation formula (nimodipine method: [(pretreatment score − posttreatment score) ÷ pretreatment score] × 100%.

### 2.2 Data source

Database Search: PubMed, Cochrane Library, AMED, EMbase, World SciNet, Nature, Science online and China National Knowledge Infrastructure, China Biomedical Literature CD-ROM Database, China Resources Database. Search for clinical research literature on TCM DN proteinuria published in domestic and foreign biomedical journals from the establishment of the library to September 2019. Based on the standards of the Cochrane Collaboration Workbook of the International Evidence-Based Medicine Center, a manual and computer-based method will be used to conduct related literature searches. The search terms include: diabetic nephropathy or diabetic kidney disease or diabetic proteinuria, Chinese medicine, traditional Chinese medicine, proprietary Chinese medicine, Chinese herbal medicine. The complete PubMed search strategy is summarized in Table 1.

**Table 1:**
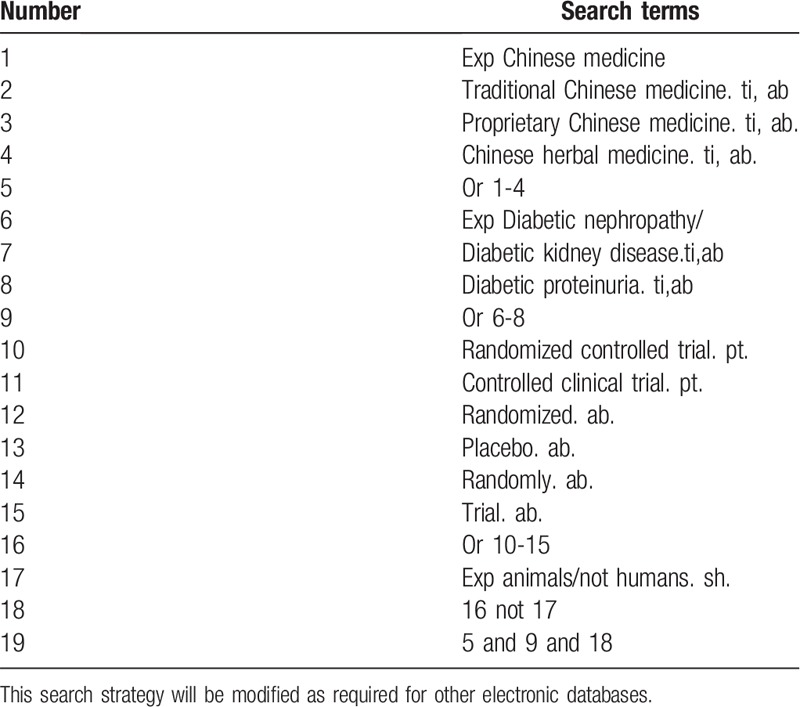
Search strategy used in PubMed database.

### 2.3 Data collection and analysis

#### 2.3.1 Selection of studies

Two investigators used EndnoteX7 software to conduct a preliminary assessment of the title and abstract of each document in the database based on the established criteria for inclusion in the study to select eligible studies. After a preliminary assessment, the full text of the selected literature would be evaluated, and the uncontrolled study, no randomization, inconsistent evaluation criteria, and similar data would be excluded. Any differences in screening that occurred during the screening study would be discussed to get consensus, if it still can not be resolved, then the third author would be intervened.

#### 2.3.2 Data extraction and management

**Figure 1:**
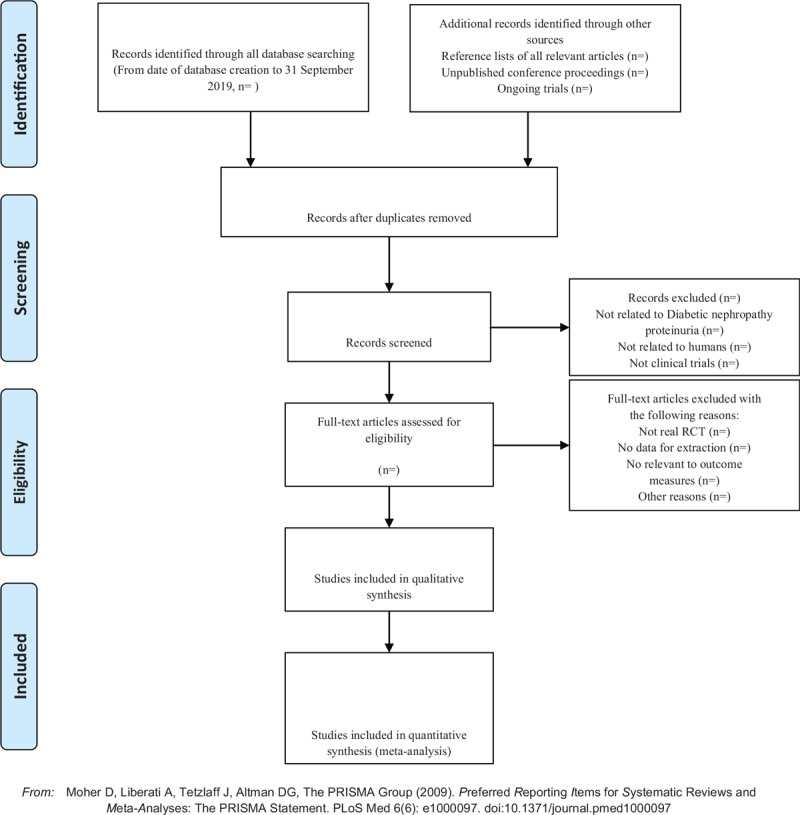
The PRISMA flow chart. PRISMA = preferred reporting items for systematic review and meta-analysis.

Two investigators independently extracted information from the included literature. The extracted content includes research design, random hiding and blinding, basic information of the included cases, intervention methods, observation indicators, and test results of the treatment group and the control group. The extracted literature data will be filled in a unified data statistics table. For studies that provide baseline and posttreatment data, we will estimate the change values by the method recommended by Cochrane. The details of selection process will be shown in the PRISMA flow chart (Fig. 1).

#### 2.3.3 Assessment of risk of bias in included studies

Two investigators will independently evaluate the methodological quality of the included literature by using the Cochrane Collaboration's ROB tool which includes whether the random method is correct, whether blinding is used, whether it is hidden, whether it is lost or quit, whether it uses intent-to-treat analysis, whether the data results are accurate, and other risks of bias. According to the relevant standards in the Cochrane Intervention System Evaluation Manual, it will be divided into low-risk, high-risk, and unclear.

#### 2.3.4 Dealing with missing data

In the event of data loss during the screening and extraction of literature data, first, we will actively investigate the cause of data loss. Then we will contact the experimental research author by telephone, mail, and so on to achieve the purpose of supplementing the missing data. If the lost data cannot be retrieved, we will only extract and analyze the useful data, besides, we will indicate the situation.

#### 2.3.5 Statistical analysis

The numerical variable will be expressed as the normalized mean difference (standardized mean difference) with a confidence interval of 95%. The heterogeneity of each pairwise comparison will be tested by the Chi-square test (test level *α* = 0.1). If there is no heterogeneity, a fixed effect model will be used. If there is significant heterogeneity between a group of studies, we will explore the reasons for the existence of heterogeneity from various aspects such as the characteristics of the subjects and the degree of variation of the interventions. Sensitivity analysis or meta-regression and subgroup analysis to explore possible sources of heterogeneity when necessary. We will use qualitative analysis of the funnel plot and graph symmetry to assess publication bias. Quantitative methods such as Begg testing and Egger testing will be used to help assess publication bias in the application.

#### 2.3.6 Assessment of heterogeneity

If there is significant heterogeneity between a group of studies, we will explore the reasons for the existence of heterogeneity from various aspects such as the characteristics of the subjects and the degree of variation of the interventions. Sensitivity analysis or subgroup analysis is performed as necessary to explain heterogeneity.

#### 2.3.7 Assessment of publication bias

The forest map and funnel plot were drawn and analyzed using Rev Man5.3 software, and the funnel plot was used to analyze potential publication bias.

#### 2.3.8 Grading the quality of evidence

The quality of evidence for the main outcomes will also be assessed with the grading of recommendations assessment approach. The evaluation included bias risk; heterogeneity; indirectness; imprecision; publication bias. And each level of evidence will be made “very low,” “low,” “moderate,” or “high” judgment.

## 3 Discussion

At present, there are vast therapies in treating DN proteinuria; however, the efficacy is still unsatisfactory due to the particularity of the anatomical structure of kidney.^[21]^ Studies have shown that drug intervention protect the renal interstitial and glomeruli to a certain extent, but there is no single drug can continue to significantly ameliorate all symptoms of DN proteinuria patients.^[22,23]^ TCM has a profound theoretical foundation and abundant clinical experience in the treatment of DN proteinuria. TCM therapy mainly achieves therapeutic effects by stimulating the body's righteousness and regulating the balance of qi and blood. In recent years, TCM therapy has been widely used in clinical trials of DN proteinuria. Recent studies have shown that TCM can alleviate the pain caused by DN proteinuria to a certain extent and improve the quality of lives of patients.^[24]^

Although abundant studies have evaluated the effectiveness of TCM in treating DN proteinuria, evaluation and comparison between various treatments are still insufficient. To the best of our knowledge, a systematic review and meta-analysis have not been used in recent years to compare the effectiveness of TCM in the treatment of DN proteinuria. The results of meta-analysis can provide a possible ranking for TCM treatment of DN proteinuria. We hope that the results will provide clinicians with the best options for treating DN proteinuria and provide them with research directions. Due to the limited number of relevant high-quality studies and the few sample size included, the strength of the arguments of the conclusions is to some degree limited. Therefore, we hope that more large-scale, high-quality RCTs should be necessary in the future. Besides, improving the quality of the original research and conducting high-quality multicenter RCTs to explore the clinical efficacy of TCM treatment of CP/CPPS is also indispensable, through which could make the conclusion more objective and reasonable.

## Author contributions

**Data curation:** Lin Wang.

**Formal analysis:** Ya-Hui Wang.

**Funding acquisition:** Xian-Hui Zhang.

**Project administration:** Xue-Lian Yang.

**Resources:** Xue-Lian Yang.

**Software:** Hui-Li Wei.

**Supervision:** Hui-Li Wei, Zhi-Chao An, Yan Guo, Hong-Fang Liu.

**Validation:** Zhi-Chao An, Bo-Rui Yu.

**Visualization:** Bo-Rui Yu, Da-Qing Du.

**Writing – original draft:** Da-Qing Du.

**Writing – review and editing:** Hong-Fang Liu.
